# Heart rate and respiratory rate in predicting risk of serious bacterial infection in febrile children given antipyretics: prospective observational study

**DOI:** 10.1007/s00431-023-04884-7

**Published:** 2023-03-03

**Authors:** Stefanie Wittmann, Rikke Jorgensen, Rianne Oostenbrink, Henriette Moll, Jethro Herberg, Mike Levin, Ian Maconochie, Ruud Nijman

**Affiliations:** 1 Department of Paediatric Emergency Medicine, Division of Medicine, St. Mary’s Hospital–Imperial College NHS Healthcare Trust, London, UK; 2 Department of General Paediatrics, Erasmus MC–Sophia Children’s Hospital, Rotterdam, The Netherlands; 3 Faculty of Medicine, Department of Infectious Diseases, Section of Paediatric Infectious Diseases, Imperial College London, London, UK; 4 Centre for Paediatrics and Child Health, Imperial College London, London, UK

**Keywords:** Fever, Child, Serious bacterial infection, Antipyretics, Vital signs

## Abstract

Clinical algorithms used in the assessment of febrile children in the Paediatric Emergency Departments are commonly based on threshold values for vital signs, which in children with fever are often outside the normal range. Our aim was to assess the diagnostic value of heart and respiratory rate for serious bacterial infection (SBI) in children after temperature lowering following administration of antipyretics. A prospective cohort of children presenting with fever between June 2014 and March 2015 at the Paediatric Emergency Department of a large teaching hospital in London, UK, was performed. Seven hundred forty children aged 1 month–16 years presenting with a fever and ≥ 1 warning signs of SBI given antipyretics were included. Tachycardia or tachypnoea were defined by different threshold values: (a) APLS threshold values, (b) age-specific and temperature-adjusted centiles charts and (c) relative difference in *z*-score. SBI was defined by a composite reference standard (cultures from a sterile site, microbiology and virology results, radiological abnormalities, expert panel). Persistent tachypnoea after body temperature lowering was an important predictor of SBI (OR 1.92, 95% CI 1.15, 3.30). This effect was only observed for pneumonia but not other SBIs. Threshold values for tachypnoea > 97th centile at repeat measurement achieved high specificity (0.95 (0.93, 0.96)) and positive likelihood ratios (LR + 3.25 (1.73, 6.11)) and may be useful for ruling in SBI, specifically pneumonia. Persistent tachycardia was not an independent predictor of SBI and had limited value as a diagnostic test.

*  Conclusion*: Among children given antipyretics, tachypnoea at repeat measurement had some value in predicting SBI and was useful to rule in pneumonia. The diagnostic value of tachycardia was poor. Overreliance on heart rate as a diagnostic feature following body temperature lowering may not be justified to facilitate safe discharge.

**Table:** 

**What is Known:** *• Abnormal vital signs at triage have limited value as a diagnostic test to identify children with SBI, and fever alters the specificity of commonly used threshold values for vital signs.* *• The observed temperature response after antipyretics is not a clinically useful indicator to differentiate the cause of febrile illness.*	
**What is New:** *• Persistent tachycardia following reduction in body temperature was not associated with an increased risk of SBI and of poor value as a diagnostic test, whilst persistent tachypnoea may indicate the presence of pneumonia.*

**Supplementary Information:**

The online version contains supplementary material available at 10.1007/s00431-023-04884-7.

## Introduction

Fever constitutes one of the most common reasons for paediatric emergency presentations accounting for up to 20% of consultations [1–3]. Vaccination programmes have reduced the incidence of vaccine preventable serious bacterial infection (SBI), especially sepsis and meningitis [4]. Nowadays, an SBI is diagnosed only in a minority of febrile children (7–10%), with pneumonia and urinary tract infections (UTI) being the most frequent [5, 6]. Most children with fever suffer from self-limiting viral illnesses, and identifying those who need further investigations and antibiotic treatment for SBI remains a diagnostic challenge.

Most clinical algorithms are based on specific thresholds for abnormal vital sign parameters such as the age-specific cut-off values published by the Advanced Life Support Group (APLS) [7]. In febrile children, heart and respiratory rates are often elevated and outside the normal range [8–11]. Temperature-dependent, age-specific centile charts for heart and respiratory rate have been developed to account for this effect of fever on vital signs [12, 13].

Antipyretics (paracetamol or ibuprofen) are frequently given to febrile children in hospital prior to evaluation, especially when abnormal heart and respiratory rates are recorded at triage which are categorised as a ‘warning sign’ based on NICE guidelines for managing febrile children ≤ 5 years [14]. It is a common practice to perform serial vital sign measurements to demonstrate a lowering effect on heart and respiratory rate following reduction in fever. However, previous research showed that temperature response to paracetamol was not a clinically useful indicator by which to differentiate the cause of febrile illness [15, 16].

There are few existing studies assessing the value of repeat vital sign measurements in predicting SBI and whether concomitant reductions in heart and respiratory rates are associated with a decreased risk of SBI. In this prospective cohort study, we investigate the value of serial heart and respiratory rate measurements following reduction in body temperature after administration of antipyretics and its association with the risk of having SBI.

## Methods

### Design, setting and participants

This prospective observational study (Infections in Children in the Emergency Department (ICED)—study) was conducted at the paediatric accident and emergency department (PED) at St. Mary’s Hospital, Imperial College NHS Healthcare Trust, London, UK [17]. The PED is a large tertiary inner-city department which sees about 27,000 children per year.

All children aged 1 month to 16 years presenting to the PED between June 2014 and March 2015 with fever and ≥ 1 amber or red warning sign(s) according to NICE Fever in under 5 s: assessment and management [14] were eligible. Fever was defined:

1. Axillary or tympanic temperature of ≥ 38.0 °C at triage
2. Fever as a discriminator in the Manchester Triage System (MTS)
3. Fever as a reason for GP referral to the PED
4. Fever ≥ 38.0 °C measured at home in the previous 24 h

Children triaged under the ‘immediate’ (red) category for emergency treatment (MTS) were excluded (treatment based on protocols for life threatening conditions). The following additional exclusion criteria were applied: (a) complex medical history (underlying medical problem requiring ≥ 2 annual specialist visits) [18], (b) not waited to be seen, (c) discharged to the urgent care centre without being seen in PED, (d) non-UK residents (determining a final outcome not possible), (e) all data for visit missing, (f) overruling of fever or warning signs as a presenting problem by the physician with the patient actively deemed non-eligible for the study, (g) patient did not consent to participation in embedded biomarker study.

Analysis was confined to children with at least one repeat set of observations consisting of either respiratory rate (RR) or heart rate (HR) and available temperature and time of measurement and was performed separately for RR and HR. PED standards dictate that abnormal vital signs are repeated within 60 min. A senior clinician may discharge the patient before this time. The last available set of repeat vital signs was used for each child. Measurements beyond the 3rd repeat were not included as these represent a minority of children with a prolonged stay in the PED (delayed transfer or resuscitation). Analysis of HR was restricted to children from 3 months to 10 years (centile charts not derived outside this range).

### Data collection

Information was collected prospectively and recorded in electronic patient records using a predefined integrated data collection form. Standardised triage data according to the MTS and warning signs of fever (NICE) were recorded at the time of triage [14]. Vital signs were measured by a nurse at triage (baseline) with repeat measurements as per the *Advanced Paediatric Life Support* (APLS) standard [7]. HR was measured through pulse oximetry in beats/minute, body temperature in °C with a tympanic or axillary thermometer and RR in breaths/minute by counting breaths for 60 s. Data on diagnostics, interventions, treatment and discharge were recorded in a data entry form and clinical details extracted from medical records.

According to local standards, patients were given either paracetamol (first line) or ibuprofen (second line) orally according to the British National Formulary (BNF) [19] if not given within 4 h at home. Children who received an antipyretic at home were eligible for receiving the antipyretic not yet given.

### Outcome measures

A final diagnosis of SBI was based on a composite reference standard combining positive cultures from a sterile site (blood, urine, CSF), available microbiology and virology results, radiological abnormalities and consensus diagnosis by an expert panel [5, 17]. SBI was further categorised into pneumonia and other SBIs (meningitis, septicaemia, UTI, cellulitis, osteomyelitis, septic arthritis) [5]. A diagnosis of pneumonia was supported by radiographic evidence of consolidation or effusion determined by a paediatric radiologist. Patients were followed up via telephone for 1 week after PED attendance and a final outcome coded by independent members of the research team, considering data from all PED visits within a 5-day period including admissions.

### Statistical analysis

The number of children with tachycardia or tachypnoea was defined using different threshold values for HR and RR at baseline and repeat measurement: (a) APLS threshold values [7], (b) HR or RR > 90th centile on age-specific temperature-adjusted centile charts and (c) *z*-score difference between corresponding *z*-scores at baseline and repeat measurement [12, 13]. Each HR or RR was transferred into a *z*-score considering body temperature and age. The *z*-score difference was calculated by subtracting the *z*-score following body temperature lowering from the *z*-score at baseline. We investigated the change in number of children classifying as tachycardic or tachypneic by APLS definition and changes in allocated age-specific and temperature-dependent centiles for HR and RR as measured by differences in *z*-scores. Diagnostic performance of repeat vital signs was assessed by odds ratios (OR) derived from logistic regression analyses adjusting for potential confounders. Age (4 standard categories), gender, *z*-score at baseline, time from triage to repeat vital sign measurement (mins), bronchodilator treatment and dehydration were considered as potential confounders. Sensitivity, specificity, positive and negative predictive value and positive and negative likelihood ratios for different threshold values for tachycardia and tachypnoea were calculated. The discriminative ability was expressed by the area under the receiver operating characteristics curve (ROC). We used a multinominal regression model and relative risk ratios (RRR) to identify if the predictive value differed by type of SBI. Stata version 15.1 was used for all statistical analysis.

An SBI prevalence of 7% has been previously published [17, 20], and a conservative sample size of *n* = 700 was estimated to achieve 12 events per predictor variable (EPP) based on inclusion of 4 parameters.

## Results

**Fig. 1:**
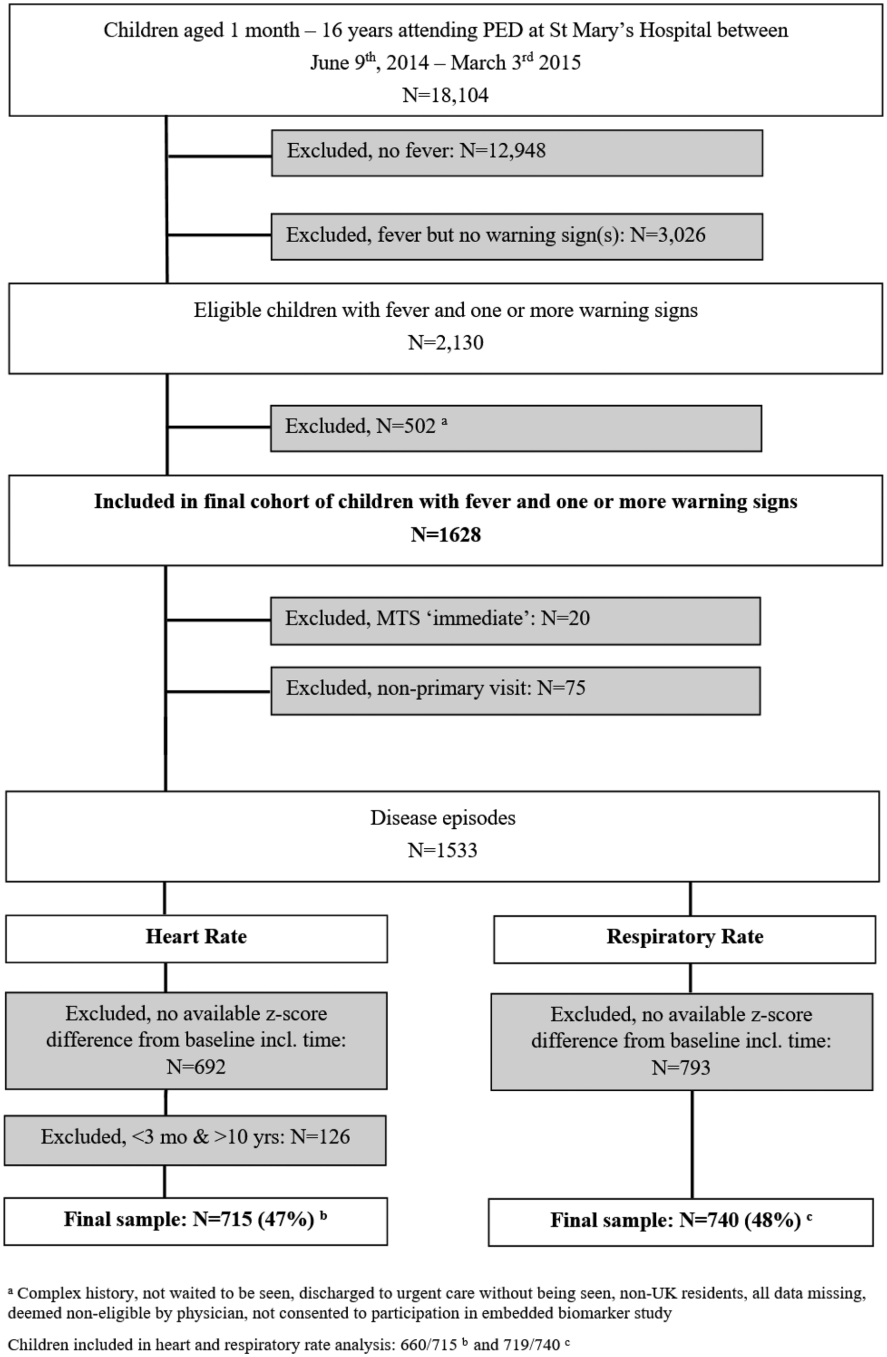
Selection of study sample

1628/2130 children attending PED with fever and ≥ 1 warning signs were eligible (Fig. 1). The final data set for analysis of HR comprised 715 and RR 740 children after excluding high acuity patients (*N* = 20), repeat presentations (*N* = 75), children without repeat observations (HR *N* = 692 and RR *N* = 793) and children outside the age range of available HR centile charts (*N* = 126). Characteristics of included and excluded children are shown in Online Resource 1.

### Study cohorts

Median age of included children was 2.2 years (IQR 1.3–4.5). The prevalence of SBI was 8.8% (*n* = 70) with pneumonia (3.4%), UTI (1.8%) and other SBIs (3.1%) the most frequent infections (Table 1).

**Table 1:** Clinical and demographic characteristics of the study population

		**Total** ***N* (%)**	**No SBI** ***N* (%)**	**SBI** ***N* (%)**
		795 (52)	725 (91)	70 (8.8)
**Age (years)**	Median (IQR)	2.2 (1.3–4.5)	2.2 (1.2–4.4)	2.6 (1.4–5.4)
	1 month– < 1 year	130 (16)	118 (16)	12 (17)
	1– < 2 years	227 (28)	212 (29)	15 (21)
	2– < 5 years	263 (33)	239 (33)	24 (34)
	5– < 16 years	175 (22)	156 (21)	19 (27)
**Gender**	Male	437 (55)	405 (56)	32 (46)
**Triage urgency**^a^	Very urgent	448 (56)	412 (57)	36 (51)
	Urgent	138 (17)	123 (17)	15 (21)
	Standard	209 (26)	190 (26)	19 (27)
	Non-urgent	-	-	-
**Body temperature (°C)**	Median (IQR)	38.5 (38.0–39.1)	38.5 (38.0–39.0)	38.8 (38.0–39.5)
**SpO2**^b^	< 94%	29 (3.6)	27 (3.7)	2 (2.9)
**CRT**^b^	> 2 s	12 (1.5)	12 (1.5)	-
**Pain at triage**		77 (10)	69 (9.5)	8 (11)
**Distress at triage**		107 (13)	100 (14)	7 (10)
**Dehydration at triage**^b^		14 (1.8)	10 (1.4)	4 (5.7)
**Post-ictal following seizure**		31 (3.9)	28 (3.9)	3 (4.3)
**Bronchodilator treatment**^c^		113 (14)	104 (14)	9 (13)
**Administration of antipyretics**^d^	None	16 (2.0)	14 (1.9)	2 (2.8)
	Yes	489 (87)	634 (87)	55 (78)
	Unknown	90 (11)	77 (11)	13 (18)
	Triage	537 (67)	494 (68)	43 (61)
	Home^e^	414 (52)	386 (53)	28 (40)
**Type of antipyretic**^b^	Paracetamol	177 (22)	162 (22)	15 (21)
	Ibuprofen	25 (3.1)	23 (3.2)	2 (2.9)
	Combination	346 (43)	320 (44)	26 (37)
**Time in department (min)**	Median (IQR)	197 (153–232)	192 (149–228)	233 (199–240)
**Referral category**^b^	Self-referred	662 (83)	602 (83)	60 (86)
	GP referral	18 (2.3)	14 (1.9)	4 (5.7)
	Ambulance	25 (3.1)	24 (3.3)	1 (1.4)
	Other	71 (8.9)	83 (11)	5 (7.1)
**SBI**		70 (8.8)	-	70 (8.8)
	Pneumonia	27 (3.4)	-	27 (3.4)
	Sepsis/meningitis	4 (0.5)	-	4 (0.5)
	UTI	14 (1.8)	-	14 (1.8)
	Other SBI	25 (3.1)	-	25 (3.1)
**Disposition**	Discharged	602 (76)	586 (81)	21 (30)
	Admitted	184 (23)	138 (19)	46 (66)
	PICU	3 (0.4)	1 (0.1)	2 (2.9)
	Died	1 (0.1)	-	1 (1.4)

*SpO2* saturation of peripheral oxygen, *CRT* capillary refill time, *UTI* urinary tract infection, *IQR* interquartile range

^a^Triage category MTS = 1 (emergent) excluded

^b^Missing values: SpO2 *n* = 13, CRT *n* = 131, dehydration *n* = 5, type of antipyretic *n* = 323, referral category *n* = 2

^c^Salbutamol administered via any route (spacer, nebuliser, IV)

^d^Paracetamol or ibuprofen administered via any route (oral, rectal, IV)

^e^Unknown *n* = 319, missing *n* = 4

### Frequency of tachycardia and tachypnoea for different thresholds

Following lowering of body temperature, APLS thresholds identified 30% of children as tachycardic versus 13% using the 90th centile on temperature-adjusted centile charts. Thirty-four percent of children remained tachypnoeic after temperature lowering applying APLS thresholds versus 15% using centile charts (Table 2). Additional values for vital signs at different repeat measurements are given in Online Resources 2 and 3.

**Table 2:** Median heart rate and respiratory rate, body temperature and proportion tachycardic or tachypnoeic at baseline and at repeat measurement

	**Baseline**	**Last available repeat**		**Baseline**	**Last available repeat**
***N* (%)**	715 (100)	715 (100)	***N* (%)**	740 (100)	740 (100)
**HR (beats/min)**^a,b^	155 (141–168)	134 (121–148)	**RR (breaths/min)**^**a**^	33 (28–42)	30 (25–36)
*3 months*– < *1 year*	168 (157–177)	147 (135–158)	*1 month–* < *1 year*	44 (38–52)	40 (34–44)
*1*– < *2 years*	162 (152–176)	141 (132–155)	*1–* < *2 years*	36 (31–44)	32 (28–37)
*2*– < *5 years*	152 (143–162)	132 (121–144)	*2–* < *5 years*	32 (28–40)	28 (26–32)
*5*– < *11 years*	135 (126–142)	115 (105–128)	*5–* < *16 years*	26 (22–28)	24 (22–26)
**Body temperature**^a^ **(°C)**	38.5 (38.0–39.2)	37.4 (36.9–37.8)	**Body temperature**^a^ **(°C)**	38.5 (38.0–39.1)	37.4 (36.9–37.8)
***Z*-score**^a^	0.57 (0.01–1.14)	0.34 (-0.27–1.00)	***Z*-score**^a^	0.46 (0.01–1.05)	0.50 (0.00–1.00)
***Z*-score difference**^a^	N/A	-0.23 (-0.81–0.39)	***Z*-score difference**^a^	N/A	−0.03 (−0.55–0.45)
**Tachycardia (APLS)**	574 (80%)	214 (30%)	**Tachypnoea (APLS)**	431 (58%)	253 (34%)
**Tachycardia (> 90th centile)**	133 (18%)	93 (13%)	**Tachypnoea (> 90th centile)**	146 (20%)	112 (15%)
**Time from triage to repeat (mins)**^a^	N/A	125 (90–165)	**Time from triage to repeat (mins)**^a^	N/A	125 (90–169)

^a^Median (IQR)

^b^Children age < 3 months and > 11 years excluded (centile charts for HR not available)

### Association between tachycardia and tachypnoea at repeat measurement and SBI for different thresholds

Children diagnosed with any SBI were significantly more likely classified as tachypnoeic on repeat measurement based on either APLS threshold values (adjusted OR 1.92, 95% CI 1.12, 3.30, *p* = 0.018) or a RR > 90th centile on temperature-adjusted centile charts (adjusted OR 2.40, 95% CI 1.21, 4.75, *p* = 0.012). *Z*-score difference was not significant in the unadjusted model (OR 1.29, 95% CI 0.97, 1.72, *p* = 0.080) for SBI. A significant association after adjusting for baseline *z*-score may be explained through a negative correlation between baseline *z*-score and *z*-score difference (Spearman’s rank correlation coefficient, −0.54, *p* < 0.0001). Children classified as tachycardic on repeat measurement by any threshold value were not significantly more likely diagnosed with an SBI (Table 3).

**Table 3:** Association between tachycardia and tachypnoea at repeat measurements for different threshold values and SBI

			**OR**	**(95% CI)**	*p*-value*	**Adjusted OR**^**a**^	**(95% CI)**	*p*-value*
**Heart rate**								
	**No SBI** (*N* = 658)	**SBI** (*N* = 57)						
**Tachycardia (APLS)**	527 (80)	47 (82)	0.99	(0.55, 1.80)	0.986	0.99	(0.53, 1.86)	0.982
**Tachycardia (> 90th centile)**	88 (13)	5 (9)	0.62	(0.24, 1.60)	0.326	0.59	(0.22, 1.57)	0.291
***Z*-score difference**	−0.23 (−0.81–0.37)	−0.21 (−0.75–0.42)	1.03	(0.79, 1.33)	0.842	1.04	(0.76, 1.42)	0.801
**Respiratory rate**								
	**No SBI** (*N* = 673)	**SBI** (*N* = 67)						
**Tachypnoea (APLS)**	222 (33)	31 (47)	1.75	(1.05, 2.90)	0.029	1.92	(1.12, 3.30)	0.018
**Tachypnoea (> 90th centile)**	95 (14)	17 (25)	2.07	(1.14, 3.74)	0.016	2.40	(1.21, 4.75)	0.012
***Z*-score difference**	−0.04 (−0.56–0.43)	0.17 (−0.46–0.64)	1.29	(0.97, 1.72)	0.079	1.56	(1.11, 2.19)	0.011

^*^Wald test

^a^Adjusted for gender, bronchodilators, *z*-score at baseline

Categorised into pneumonia and other SBIs, persistent tachypnoea was strongly related to pneumonia (RRR 7.11, 95% CI 2.83, 17.87, *p* < 0.0001 and RRR 5.65, 95% CI 2.57, 12.39, *p* < 0.0001, respectively) though their value for the prediction of other SBIs or the use of *z*-score difference in predicting any SBI appeared to be limited (Table 4). Persistent tachycardia was not useful for predicting pneumonia or other SBIs.

**Table 4:** Analysis between individual predictors and SBI stratified by type of infection for respiratory rate (relative risk ratio (RRR) from multinomial regression)

	**RRR**	**(95% CI)**	***p*-value**		**RRR**	**(95% CI)**	***p*-value**
**Tachypnoea (APLS)**				**Tachycardia (APLS)**			
Pneumonia	7.11	(2.83, 17.87)	< 0.0001	Pneumonia	0.83	(0.32, 2.12)	0.692
Other SBI	0.68	(0.32, 1.41)	0.298	Other SBI	1.12	(0.53, 2.34)	0.765
**Tachypnoea (> 90th centile)**				**Tachycardia (> 90th centile)**			
Pneumonia	5.65	(2.57, 12.39)	< 0.0001	Pneumonia	0.29	(0.04, 2.21)	0.235
Other SBI	0.68	(0.23, 1.94)	0.467	Other SBI	0.86	(0.30, 2.51)	0.788
***Z*-score difference**				***Z*-score difference**			
Pneumonia	1.28	(0.83, 1.98)	0.266	Pneumonia	1.18	(0.79, 1.76)	0.417
Other SBI	1.30	(0.90, 1.86)	0.154	Other SBI	0.93	(0.67, 1.30)	0.690

Constraints due to low event numbers per stratum precluded further adjustment for covariates

### Diagnostic performance and discriminative ability of change in HR and RR for different threshold values

APLS thresholds for tachypnoea at repeat measurement had poor sensitivity (0.46, 95% CI 0.34, 0.59) and specificity (0.67, 95% CI 0.63, 0.71) with moderate specificity for tachypnoea using cut-off values > 90th centile on centile charts (0.86, 95% CI 0.83, 0.88). Threshold values > 97th centile achieved high specificity (0.95, 95% CI 0.93, 0.96) and positive likelihood ratios (LR + 3.25. 95% CI 1.73, 6.11) and were useful for ruling in SBI. Persistent tachypnoea showed poor discriminative ability at repeat measurement following change in body temperature for all assessed thresholds (ROC 0.48–0.57).

Persistent tachycardia (APLS thresholds) had poor sensitivity (0.30, 95% CI 0.18, 0.43) at repeat measurement. Specificity for centile cut offs > 97th was high (0.96, 95% CI 0.94, 0.97), but low positive likelihood ratios make tachycardia according to either threshold less useful for ruling in SBI. Persistent tachycardia at repeat measurement after body temperature lowering had poor discriminative ability for the diagnosis of SBI regardless of which threshold was used (range ROC 0.48–0.52) (Table 5).

**Table 5:** Diagnostic performance and discriminative ability of different thresholds for heart rate and respiratory rate at repeat measurement

	**Sensitivity**	**Specificity**	**PPV**	**NPV**	**LR + **	**LR-**	**ROC**
**Tachycardia APLS**							
	0.30 (0.18, 0.43)	0.70 (0.66, 0.73)	0.08 (0.05, 0.12)	0.92 (0.90, 0.94)	1.00 (0.66, 1.51)	1.00 (0.84, 1.19)	0.50 (0.44, 0.56)
**Tachycardia centile thresholds**							
**50th**	0.67 (0.53, 0.79)	0.37 (0.33, 0.41)	0.08 (0.06, 0.11)	0.93 (0.89, 0.96)	1.05 (0.87, 1.28)	0.91 (0.62, 1.33)	0.52 (0.45, 0.58)
**75th**	0.33 (0.21, 0.47)	0.64 (0.60, 0.67)	0.07 (0.04, 0.11)	0.92 (0.89, 0.94)	0.92 (0.63, 1.35)	1.04 (0.86, 1.27)	0.49 (0.42, 0.55)
**90th**	0.09 (0.03, 0.19)	0.87 (0.84, 0.89)	0.05 (0.02, 0.12)	0.92 (0.89, 0.94)	0.66 (0.28, 1.55)	1.05 (0.97, 1.15)	0.48 (0.44, 0.52)
**97th**	0.03 (0.00, 0.12)	0.96 (0.94, 0.97)	0.07 (0.01, 0.22)	0.92 (0.90, 0.94)	0.82 (0.20, 3.37)	1.01 (0.96, 1.06)	0.50 (0.47, 0.52)
***Z*-score difference**							
	-	-	-	-	-	-	0.50 (0.41, 0.58)
**Tachypnoea APLS**							
	0.46 (0.34, 0.59)	0.67 (0.63, 0.71)	0.12 (0.08, 0.17)	0.93 (0.90, 0.95)	1.4 (1.06, 1.86)	0.80 (0.64, 1.01)	0.57 (0.50, 0.63)
**Tachypnoea centile thresholds**							
**50th**	0.82 (0.71, 0.90)	0.25 (0.21, 0.29)	0.10 (0.07, 0.13)	0.93 (0.89, 0.96)	1.1 (0.97, 1.24)	0.71 (0.42, 1.21)	0.54 (0.49, 0.58)
**75th**	0.43 (0.31, 0.56)	0.61 (0.58, 0.65)	0.10 (0.07, 0.14)	0.92 (0.89, 0.94)	1.12 (0.84, 1.5)	0.92 (0.74, 1.15)	0.52 (0.46, 0.59)
**90th**	0.25 (0.15, 0.37)	0.86 (0.83, 0.88)	0.15 (0.09, 0.23)	0.92 (0.90, 0.94)	1.8 (1.15, 2.82)	0.87 (0.75, 1.00)	0.56 (0.50, 0.61)
**97th**	0.16 (0.85, 0.27)	0.95 (0.93, 0.96)	0.24 (0.13, 0.40)	0.92 (0.90, 0.94)	3.25 (1.73, 6.11)	0.88 (0.78, 0.98)	0.56 (0.51, 0.60)
***Z*-score difference**							
					-	-	0.55 (0.46, 0.63)

*PPV* positive predictive value, *NPV* negative predictive value, *LR* likelihood ratio, *ROC* area under the receiver operating characteristics curve

## Discussion

### Principal findings

Persistent tachycardia following reduction in body temperature was not associated with an increased risk of SBI in children presenting to the PED with fever and ≥ 1 NICE warning signs. The presence of tachycardia on repeat measurement had low sensitivity and was of limited value as a rule in or a rule out feature of SBI. Tachypnoea (APLS or temperature-adjusted centile charts) at repeat measurement had some value in predicting SBI and was useful to rule in pneumonia, especially for threshold values > 97th centile (LR + 3.25).

### Comparison with previous research

Abnormal vital signs are used to identify children at risk of SBI. During episodes of fever, the normal range for HR and RR often differs from APLS thresholds, and the application of temperature-adjusted centiles in our study underlined the impact fever has on vital signs [14, 15]. In a previous study, RR values based on temperature-adjusted centiles performed better than APLS thresholds in predicting the risk of pneumonia with values > 97th centile highly specific and useful as a rule in feature, similar to our study [13]. Interestingly, this effect was not replicated for children with sepsis and APLS-based definitions for tachycardia outperformed the temperature-adjusted centiles [21]. In this study, tachycardia was not an independent predictor of SBI, irrespective of temperature lowering and thresholds used. Similar to our findings, the utility of tachycardia as a diagnostic tool was found to be limited in previous studies [20–22]. It is common practice to administer antipyretics to febrile children, and 67% received additional full or partial doses at triage. Less than 2% had no evidence of antipyretic exposure. A variety of timing and dosing regimens prohibited further stratification by type of antipyretic. There is currently little evidence to suggest the effect of different antipyretics on fever differs significantly [23, 24].

### Strengths and weaknesses

One strength of this study is the large cohort of febrile children representing a broad population of children with fever and warning signs, an everyday clinical dilemma in acute care. Data collection was conducted prospectively as part of clinical care increasing generalisability. Healthcare professionals were unaware of the study question limiting the potential for observer bias. The prevalence of SBI in children with and without documented repeat vital signs was not significantly different (8% vs 7%,Online Resource 1), and analysed patients should adequately represent an important group with higher diagnostic uncertainty. Reference investigations are not routinely performed on all children leading to potential verification bias. Standard diagnostic tests are imperfect, and it may not be possible to differentiate between bacterial and viral pneumonia. We attempted to limit this impact by applying a composite reference standard and use of an expert panel allowing inclusion of additional information including a follow-up period [25]. Measurement of predictor variables within the clinical setting may be prone to error and interrater variability which aligns with routine practice where the predictors will be used. Although APLS protocols provide a standard of measurement, counting breaths is known to lack accuracy compared to more objective devices and varies by level of expertise [26]. The low number of events in the individual SBI outcome categories resulted in imprecise effect estimates and may have been underpowered to detect an effect for other SBIs. Low prevalence of invasive bacterial infections (IBI) and small number of children requiring admission to PICU precluded inclusion as a secondary outcome.

### Clinical and research implications

The results of this study reemphasise NICE recommendations that vital signs should not be used in isolation to guide decision-making on discharge and overreliance on HR as a diagnostic feature following body temperature lowering may not be justified. Our findings question the utility of giving antipyretics to well-appearing febrile children awaiting HR normalisation prior to discharge which might not be a cost-effective management strategy, with minimal yield of potentially missed SBI. Whilst children with tachycardia at discharge may be more likely to return, isolated tachycardia was not a useful predictor for future admission or need for significant interventions [27]. Our study showed similar results with few children requiring admission or significant intervention on return visit, whilst one child died of sepsis several hours after discharge when normal vital signs were recorded (Online Resources 4 and 5). Almost all febrile children (98%) were given antipyretics, either by parents or health professionals. Fever is a physiological mechanism to infection, and beneficial effects on the immune response have been demonstrated [28]. Although antipyretics alleviate some of the discomfort associated with fever, their near universal administration may not be a rational clinical intervention.

Whilst studies looking at scores, such as the LqSOFA [29], have shown the value of vital sign-based scores, larger studies need to be conducted in different settings and populations to evaluate the value of temperature-adjusted centile charts and incorporation of more subjective signs in prediction models.

## Supplementary Information

Below is the link to the electronic supplementary material.

Supplementary file1 (PDF 191 KB)

## Data Availability

Data available from corresponding author upon reasonable request.

## Abbreviations

APLS: *Advanced Paediatric Life Support*
CRT: Capillary refill time
CSF: Cerebrospinal fluid
EPP: Events per predictor variable
HR: Heart rate
MTS: Manchester Triage System
LR +: Positive likelihood ratio
LR-: Negative likelihood ratio
LRTI: Lower respiratory tract infection
NICE: National Institute for Health and Care Excellence
NPV: Negative predictive value
PED: Paediatric Emergency Department
PICU: Paediatric intensive care unit
PPV: Positive predictive value
ROC: Area under the receiver operating characteristics curve
RR: Respiratory rate
SBI: Serious bacterial infection
UTI: Urinary tract infection

## References

[1] Sands R, Shanmugavadivel D, Stephenson T (2012). Medical problems presenting to paediatric emergency departments: 10 years on. Emerg Med J.

[2] Armon K, Stephenson T, Gabriel V (2001). Determining the common medical presenting problems to an accident and emergency department. Arch Dis Child.

[3] Alpern ER, Stanley RM, Gorelick MH et al (2006) Epidemiology of a pediatric emergency medicine research network: the PECARN Core Data Project. http://journals.lww.com/pec-online 10.1097/01.pec.0000236830.39194.c0 17047467

[4] le Doare K, Nichols AL, Payne H (2014). Very low rates of culture-confirmed invasive bacterial infections in a prospective 3-year population-based surveillance in Southwest London. Arch Dis Child.

[5] Nijman RG, Vergouwe Y, Thompson M (2013). Clinical prediction model to aid emergency doctors managing febrile children at risk of serious bacterial infections: diagnostic study. BMJ (Online).

[6] Hagedoorn NN, Borensztajn DM, Nijman R et al (2020) Variation in antibiotic prescription rates in febrile children presenting to emergency departments across Europe (MOFICHE): a multicentre observational study. PLoS Med **7**. 10.1371/JOURNAL.PMED.1003208 10.1371/journal.pmed.1003208 PMC7444592 32813708

[7] Samuels M, Wieteska S (2016). editors. Advanced Paediatric Life Support Wiley.

[8] Davies P, Maconochie I (2009). The relationship between body temperature, heart rate and respiratory rate in children. Emerg Med J.

[9] Hanna CM, Greenes DS (2004). How much tachycardia in infants can be attributed to fever?. Ann Emerg Med.

[10] Gadomski AM, Fermutt T, Stanton B (1994). Correcting respiratory rate for the presence of fever*. J Clin Epidemiol.

[11] Heal C, Harvey A, Brown S et al (2022) The association between temperature, heart rate, and respiratory rate in children aged under 16 years attending urgent and emergency care settings. Eur J Emerg Med Published Online First 9 10.1097/MEJ.0000000000000951 10.1097/MEJ.0000000000000951 PMC9605188 35679531

[12] Thompson M, Harnden A, Perera R (2009). Deriving temperature and age appropriate heart rate centiles for children with acute infections. Arch Dis Child.

[13] Nijman RG, Thompson M, van Veen M (2012). Derivation and validation of age and temperature specific reference values and centile charts to predict lower respiratory tract infection in children with fever: prospective observational study. BMJ (Online).

[14] National Institute for Health and Clinical Excellence (2013) NICE Clinical Guideline 160: feverish illness in children. Assessment and initial management in children younger than 5 years

[15] Baker MD, Fosarelli PD, Carpenter RO (1987). Childhood fever: correlation of diagnosis with temperature response to acetaminophen. Pediatrics.

[16] Mackowiak PA (2000) Diagnostic implications and clinical consequences of antipyretic therapy. Clin Infect Dis 31 10.1086/317512 10.1086/317512 11113028

[17] Nijman RG, Jorgensen R, Levin M et al (2020) Management of children with fever at risk for pediatric sepsis: a prospective study in pediatric emergency care. Front Pediatr 8. 10.3389/fped.2020.548154 10.3389/fped.2020.548154 PMC7527403 33042929

[18] Simon TD, Cawthon ML, Stanford S et al (2016) Pediatric medical complexity algorithm: a new method to stratify children by medical complexity. Pediatrics 133:e1647–54. www.aappublications.org/news 10.1542/peds.2013-3875 PMC4035595 24819580

[19] Paediatric Formulary Committee (2021) BNF for Children. London: BMJ Group. Pharm Press RCPCH Public

[20] Craig JC, Williams GJ, Jones M (2010). The accuracy of clinical symptoms and signs for the diagnosis of serious bacterial infection in young febrile children: prospective cohort study of 15 781 febrile illnesses. BMJ (Online).

[21] Brent AJ, Lakhanpaul M, Ninis N (2011). Evaluation of temperature-pulse centile charts in identifying serious bacterial illness: observational cohort study. Arch Dis Child.

[22] Thompson M, Coad N, Harnden A (2009). How well do vital signs identify children with serious infections in paediatric emergency care?. Arch Dis Child.

[23] Wong T, Stang AS, Ganshorn H (2014). Combined and alternating paracetamol and ibuprofen therapy for febrile children. Evidence-Based Child Health.

[24] Paul IM, Walson PD (2021). Acetaminophen and ibuprofen in the treatment of pediatric fever: a narrative review. Curr Med Res Opin.

[25] Reitsma JB, Rutjes AWS, Khan KS (2009). A review of solutions for diagnostic accuracy studies with an imperfect or missing reference standard. J Clin Epidemiol.

[26] Lovett PB, Buchwald JM, Stürmann K et al (2005) The vexatious vital: neither clinical measurements by nurses nor an electronic monitor provides accurate measurements of respiratory rate in triage. Ann Emerg Med 45. 10.1016/j.annemergmed.2004.06.016 10.1016/j.annemergmed.2004.06.016 15635313

[27] Wilson PM, Florin TA, Huang G (2017). Is tachycardia at discharge from the pediatric emergency department a cause for concern? A nonconcurrent cohort study. Ann Emerg Med.

[28] Sullivan JE, Farrar HC (2011) Fever and antipyretic use in children. Pediatrics 127. 10.1542/peds.2010-3852 10.1542/peds.2010-3852 21357332

[29] Romaine ST, Potter J, Khanijau A et al Accuracy of a modified qSOFA score for predicting critical care admission in febrile children. http://www.publications.aap.org/pediatrics/article-pdf/146/4/e20200782/1245832/peds_20200782.pdf 10.1542/peds.2020-0782 PMC7786830 32978294

