# Supplementary material for: Heart rate and respiratory rate in predicting risk of serious bacterial infection in febrile children given antipyretics: prospective observational study
Source: Eur J Pediatr. 2023 Mar 3;182(5):2205–14. doi: 10.1007/s00431-023-04884-7 (PMC10175419; doi:10.1007/s00431-023-04884-7)
Supplement: Supplementary file 1 — Supplementary file1 (PDF 191 KB) [file 431_2023_4884_MOESM1_ESM.pdf]

Journal

The European Journal of Pediatrics

Title

Heart Rate and Respiratory Rate in predicting Risk of Serious Bacterial Infection in Febrile Children given Antipyretics: prospective observational study

Authors

S Wittmann[1] , R Jorgensen[1], R Oostenbrink[2], HA Moll [2], JA Herberg [3,4], M Levin [3,4], IK Maconochie[1] , RG Nijman[1,3,4]

Affiliation

[1] Department of Paediatric Emergency Medicine, Division of Medicine, St. Mary's hospital - Imperial College NHS Healthcare Trust, London, UK

[2] Department of General Paediatrics, Erasmus MC – Sophia Children's Hospital, Rotterdam, The Netherlands

[3] Faculty of Medicine, Department of Infectious Diseases, Section of Paediatric Infectious Diseases, Imperial College London, UK

[4] Centre for Paediatrics and Child Health, Imperial College, London, UK

Corresponding author:

R.G. Nijman, email: r.nijman@imperial.ac.uk

Online resource 1: Clinical and demographic characteristics of included and excluded children

		Total cohort N (%)	HR sample N (%)	RR sample N (%)	Incomplete records[b] N (%)
		1608 (100)	715 (44.4) [a]	740 (46.0)	772 (48.0)
**Age (Years)**	Median (IQR)	2.6 (1.4 – 5.2)	2.2 (1.3 – 4.2)	2.3 (1.3 – 4.7)	3.0 (1.6 – 5.7)
	1 month - <1 year	226 (14.0)	103 (14.4)	125 (16.9)	88 (11.4)
	1 - <2 years	405 (25.2)	219 (30.6)	202 (27.3)	167 (21.6)
	2 - <5 years	550 (34.2)	260 (36.4)	248 (33.5)	274 (35.5)
	5 - <16 years	427 (26.5)	133 (18.6)	165 (22.3)	243 (31.5)
**Gender**	Male	885 (55.0)	404 (56.5)	411 (55.5)	426 (55.2)
**Triage urgency**	Very urgent	789 (49.1)	408 (57.1)	426 (57.6)	318 (41.2)
	Urgent	279 (17.3)	124 (17.3)	130 (17.6)	135 (17.5)
	Standard	538 (33.5)	183 (25.6)	184 (24.9)	317 (41.1)
	Non-urgent	2 (0.12)	-	-	2 (0.26)
**Body temperature (°C)**	Median (IQR)	38.4 (38.0 - 39.0)	38.5 (38.0 – 39.2)	38.5 (38.0 – 39.1)	38.4 (37.9 – 38.9)
**HR (beats/min)**	Median (IQR)	149 (132 – 164)	155 (141 – 168)	154 (139 – 167)	143 (126 – 158)
**Tachycardia (APLS)**		1170 (72.7)	574 (80.3)	584 (78.9)	458 (64.3)
**Tachycardia (Centile>90th)***		230 (15.5)	133 (18.6)	123 (18.1)	90 (12.6)
**Z-score HR***	Median (IQR)	0.44 (-0.06 – 1.04)	0.57 (0.01 – 1.14)	0.55 (0.00 – 1.12)	0.36 (-0.21 – 0.90)
**RR (breaths/min)**	Median (IQR)	32 (27 - 40)	34 (28 – 41)	33 (28 – 42)	30 (26 - 36)
**Tachypnoea (APLS)**		841 (52.3)	409 (57.2)	431 (58.2)	367 (47.5)
**Tachypnoea (Centile >90th)**		229 (14.2)	138 (19.3)	146 (19.7)	75 (9.7)
**Z-score RR**	Median (IQR)	0.46 (-0.01 – 0.96)	0.46 (0.03 – 1.06)	0.59 (0.01 – 1.05)	0.46 (-0.02 – 0.88)
**SpO2**	Median (IQR)	99 (97 – 100)	99 (97 – 100)	99 (97 – 100)	99 (97 – 100)
**CRT <3 sec**		1254 (78.0)	595 (83.2)	607 (82.0)	567 (73.4)

**Pain at triage**		151 (9.4)	65 (9.1)	73 (9.9)	72 (9.3)
**Distress at triage**		194 (12.1)	100 (14.0)	91 (12.3)	83 (10.7)
**Dehydration**		19 (1.1)	11 (1.5)	11 (1.5)	4 (0.5)
**Post-seizure**		61 (3.8)	30 (4.2)	30 (4.0)	30 (4.0)
**Bronchodilator treatment**		181 (11.3)	108 (15.1)	112 (15.1)	63 (8.2)
**Administration of antipyretics**	None	21 (1.3)	11 (1.5)	14 (1.9)	5 (0.6)
	Triage	1027 (63.9)	491 (68.7)	501 (67.7)	474 (61.4)
	Home	799 (49.7)	381 (53.3)	384 (51.9)	375 (48.6)
**Disposition**	Discharged	1349 (83.9)	572 (80.0)	566 (76.5)	708 (91.7)
	Admitted	251 (15.6)	139 (19.4)	169 (22.8)	61 (7.9)
	PICU	4 (0.25)	2 (0.3)	3 (0.4)	1 (0.1)
**SBI**		115 (7.1)	57 (8.0)	67 (9.0)	42 (5.4)
	Pneumonia	42 (2.6)	23 (3.2)	27 (3.5)	15 (1.9)
	Sepsis/meningitis	7 (0.4)	2 (0.3)	4 (0.5)	2 (0.3)
	UTI	23 (1.4)	10 (1.4)	13 (1.8)	8 (1.0)
	Other SBI	43 (2.7)	22 (3.1)	23 (3.1)	17 (2.2)
**Type of visit**	Primary	1533 (95.3)	715 (100)	740 (100)	733 (94.9)
	Non-primary	75 (4.7)	-	-	39 (5.0)
**Time in Department**	Median (IQR)	174 (123 – 220)	194 (150 – 230)	199 (155 – 233)	142 (100 – 194)
**Referral category**	Self-referred	1335 (83.0)	596 (83.4)	616 (83.2)	641 (83.2)
	GP referral	42 (2.6)	17 (2.4)	17 (2.3)	21 (2.7)
	Ambulance	44 (2.7)	24 (3.4)	25 (3.4)	17 (2.2)
	Other	183 (11.3)	76 (15.1)	64 (8.6)	78 (10.1)

[a] age < 3 months & > 10 years excluded (n=126) [b] excluded from analysis RR= respiratory rate HR= heart rate

Online resource 2: Median respiratory rate (RR), body temperature and proportion tachypnoeic at baseline and at repeat measurements

	**Baseline**	**1st repeat**	**2nd repeat**	**3rd repeat**	**Last available repeat**
**N (%)**	740 (100)	693 (94)	246 (33)	85 (11)	740 (100)
**RR (breaths/min)[a]**	33 (28 – 42)	30 (26 – 37)	31 (27 - 38)	30 (26 – 39)	30 (25 – 36)
1 month - < 1 year	44 (38 – 52)	40 (34 – 46)	42 (33 – 48)	42 (38 -48)	40 (34 – 44)
1 - < 2 years	36 (31 – 44)	32 (28 – 38)	32 (30 – 39)	33 (27 – 43)	32 (28 – 37)
2 - < 5 years	32 (28 – 40)	28 (26 – 34)	30 (28 – 36)	30 (27 – 38)	28 (26 – 32)
5 - < 16 years	26 (22 – 28)	24 (22 – 28)	24 (22 – 28)	25 (22 – 28)	24 (22 – 26)
**Body temperature[a] (ºC)**	38.5 (38.0 – 39.1)	37.5 (37.0 – 38.0)	37.4 (36.9 – 38.0)	37.5 (37.1 – 38.0)	37.4 (36.9 – 37.8)
**Z-score[a]**	0.46 (0.01 – 1.05)	0.50 (0.00 – 1.00)	0.63 (0.08 – 1.17)	0.63 (0.13 – 1.36)	0.50 (0.00 – 1.00)
**Z-score difference[a]**	N/A	-0.00 (-0.47 – 0.44)	-0.06 (-0.70 – 0.47)	-0.17 (-0.64 – 0.46)	-0.03 (-0.55 – 0.45)
**Tachypnoea [b] (APLS)**	431 (58)	259 (37)	112 (45)	40 (47)	253 (34)
**Tachypnoea [b] (>90th centile)**	146 (20)	102 (14)	47 (19)	25 (29)	112 (15)
**Time from triage to repeat (mins)[a]**	N/A	97 (67 – 130)	145 (113 – 178)	184 (143 – 208)	125 (90 – 169)

[a] Median (IQR) [b] N (%)

Online resource 3: Median heart rate (HR), body temperature and proportion tachycardic at baseline and at repeat measurements

	**Baseline**	**1st repeat**	**2nd repeat**	**3rd repeat**	**Last available repeat**
**N (%)**	715 (100)	680 (95)	235 (33)	77 (11)	715 (100)
**HR (beats/min)** [a]	155 (141 – 168)	138 (125 – 151)	136 (121 – 151)	142 (128 – 154)	134 (121 – 148)
3 months -< 1year	168 (157 – 177)	150 (138 – 162)	150 (135 – 160)	156 (148 – 163)	147 (135 – 158)
1 - < 2 years	162 (152 – 176)	144 (133 – 160)	142 (134 – 155)	148 (139 – 157)	141 (132 – 155)
2 - < 5 years	152 (143 – 162)	136 (124 – 148)	135 (119 – 148)	141 (129 – 152)	132 (121 – 144)
5 - < 11 years	135 (126 – 142)	118 (108 – 132)	117 (108 – 129)	121 (108 – 138)	115 (105 – 128)
**Body temperature**[a] **(^{0}C)**	38.5 (38.0 – 39.2)	37.5 (37.0 – 38.0)	37.5 (37.0 – 38.0)	37.5 (37.1 – 38.0)	37.4 (36.9 – 37.8)
**Z-score** [a]	0.57 (0.01 – 1.14)	0.37(-0.26 – 1.02)	0.34 (-0.22 – 1.06)	0.55 (0.14 – 1.25)	0.34 (-0.27 – 1.00)
**Z-score difference**[b]	N/A	-0.19 (-0.75 – 0.35)	-0.20 (-0.96 – 0.32	-0.20 (-0.76 – 0.59)	-0.23 (-0.81 – 0.39)
**Tachycardia** [b] **(APLS)**	574 (80)	263 (39)	82 (35)	37 (48)	214 (30)
**Tachycardia** [b] **(>90th centile)**	133 (18)	105 (15)	32 (14)	15 (19)	93 (13)
**Time from triage to repeat (mins)** [a]	N/A	96 (67 – 130)	145 (112 – 178)	188 (143 – 208)	125 (90 – 165)

[a] Median (IQR)   [b] N(%)

Online resource 4: Outcome of index visit and return visit leading to admission in children discharged at their initial presentation without a final diagnosis of SBI

Age (Years)	Gender	Repeat HR	Repeat RR	Outcome index visit	Time to return visit	Outcome return visit	Diagnosis
2.1	Female	**APLS:** No tachycardia **> 90th Centile:** No tachycardia	**APLS:** No tachypnoea **> 90th Centile:** No tachypnoea	Discharged	48-72 hours	Admitted	Viral respiratory tract infection
0.7	Female	**APLS:** No tachycardia **> 90th Centile:** No tachycardia	**APLS:** No tachypnoea **> 90th Centile:** No tachypnoea	Discharged	24-48 hours	Admitted	Viral respiratory tract infection
1.1	Male	**APLS:** No tachycardia **> 90th Centile:** No tachycardia	**APLS:** Tachypnoea **> 90th Centile:** No tachypnoea	Discharged	24-48 hours	Admitted	Viral respiratory tract infection
0.9	Female	**APLS:** Tachycardia **> 90th Centile:** No tachycardia	**APLS:** Tachypnoea **> 90th Centile:** Tachypnoea	Discharged	24-48 hours	Admitted	Viral respiratory tract infection
2.2	Female	**APLS:** No tachycardia **> 90th Centile:** Tachycardia	**APLS:** No tachypnoea **> 90th Centile:** No tachypnoea	Discharged	48-72 hours	Admitted	Viral respiratory tract infection
* N= 82/725 (11%) children without SBI had at least one return visit (n=3 missing data) and no return visit required PICU.							

Online resource 5: Outcome of index visit and return visit leading to admission in children discharged at their initial presentation with a final diagnosis of SBI

Age (Years)	Gender	Repeat HR	Repeat RR	Outcome index visit	Initial diagnosis	Time to return visit	Outcome return visit	Diagnosis
1.4	Male	**APLS:** No tachycardia **> 90th Centile:** No tachycardia	**APLS:** No tachypnoea **> 90th Centile:** No tachypnoea	Discharged	Viral illness	48-72 hours	Admitted	Pneumonia
1.3	Male	**APLS:** No tachycardia **> 90th Centile:** No tachycardia	**APLS:** No tachypnoea **> 90th Centile:** No tachypnoea	Discharged	URTI	<24 hours	Died	Sepsis
1.0	Male	**APLS:** No tachycardia **> 90th Centile:** No tachycardia	**APLS:** No tachypnoea **> 90th Centile:** No tachypnoea	Discharged	URTI/tonsillitis	24-48 hours	Admitted	Periorbital cellulitis
1.8	Male	**APLS:** Tachycardia **> 90th Centile:** No tachycardia	**APLS:** Tachypnoea **> 90th Centile:** No tachypnoea	Discharged	Viral URTI	48-72 hours	Admitted	Pneumonia
* N= 8/70 (11%) children with SBI had at least one return visit (n=1 missing data). No children required admission to PICU								
